# Implementing the interRAI Check-Up Comprehensive Assessment: Facilitating Care Planning and Care Coordination during the Pandemic

**DOI:** 10.5334/ijic.7007

**Published:** 2023-04-10

**Authors:** Connie Louise Schumacher, Rebecca Correia, Sophie Hogeveen, Megan Salter, Bailey Donaldson

**Affiliations:** 1 Brock University, CA; 2 McMaster University, CA

**Keywords:** integrated care, telephone assessment, care coordination, older adults, COVID 19, community care

## Abstract

**Background:**

Long-stay home care patients are a large population of older adults with multi-morbidity and frailty. The COVID-19 pandemic posed challenges to executing care coordination and completing in-home assessments due to provincial mandates restricting in-person care. We evaluated the implementation of the interRAI Check-Up Self-Report instrument administered by phone and video.

**Methods:**

We report on a mixed-methods study, which involved the collection and analysis of survey and focus group data. Care coordinators from two regions in Ontario who had implemented the Check-Up at least once between March 2020 to September 2021 were recruited via convenience sampling.

**Results:**

A total of 48 survey respondents and 7 focus group participants consented to the study. Advantages of completing the Check-Up over the telephone or video call included: reduced travel time, reduced risk of disease transmission, familiarity with the assessment questions, and reduced time spent administering the assessment. Limitations most frequently reported were: the inability to see the living environment, hearing impairments, inability to observe non-verbal responses or cues, language barriers, difficulty building rapport, and difficulty understanding the patient.

**Conclusions:**

The Check-Up was advantageous in providing sufficient information to create a care plan when administered over the phone and by video. Implementation of the Check-Up assessment was facilitated by familiarity and alignment with other interRAI assessments. Our results indicate that population characteristics need to be taken into consideration for administration of self-report style of assessments.

## Introduction

The novel coronavirus, COVID-19, has had devastating consequences for vulnerable older adults since it was first identified in late 2019 [1,2]. Older adults continue to disproportionately suffer the most severe outcomes caused by COVID-19, including hospitalization, critical illness, intubation, stroke, and death [3,4]. Long-stay home care patients are a prevalent group of frail older adults living in the community with a high emergency department (ED) visit rate compared to those living independently without formal home care services or in long-term care [5,6,7]. In comparison to short-stay patients, long-stay home care patients typically have care needs that would require services for more than 2 months [8,9]. This population has become more complex and high needs in the past decade, with an increase in the number of home care patients who are homebound, with dependency on others, impaired cognition, and the presence of heart failure or chronic obstructive lung disease [7].

For the years preceding COVID-19, long-stay home care patients in Ontario, Canada, were assessed with the interRAI Home Care (interRAI HC), a comprehensive home care assessment, typically completed in-person [10,11]. The interRAI HC is a comprehensive, clinician-administered assessment where the assessor uses their clinical judgement to collect and reconcile clinical information from multiple sources. In the Ontario home care setting, care coordinators (regulated health professionals with nursing, physiotherapy, occupational therapy or social work designations) complete assessments with the patient and family. The interRAI HC is part of a suite of assessment instruments developed by interRAI that use common language and measures, and span the health continuum and care sectors to capture the trajectory of health, illness and care history for each patient in an integrated and consistent way.

Given the far-reaching effects of the COVID-19 pandemic, a concerted effort to reduce the spread and protect older adults was implemented in Ontario [12]. Beginning in March 2020, provincial public health directives regarding COVID-19 mandated social distancing and reduced face-to-face contact in the Home and Community Care Support Services sector [13]. Initially, a pre-existing functional assessment form served as a template for telephone calls, where changes could be documented in the static notes section of the client health record. The functional assessment is not a comprehensive assessment and does not have the functionality to electronically integrate with other existing assessments. To mitigate risk associated with in-person assessments and ensure continuity of care by completing assessments in a way more consistent with the interRAI HC previously used, the interRAI Check-Up Self-Report (CU) assessment was adopted in three regions of Ontario: Waterloo-Wellington (WW), Haldimand-Norfolk-Hamilton-Brant (HNHB), and Erie-St. Clair. The CU is a comprehensive self-report assessment that can be completed in-person, by video call or telephone. The self-report style of questions differ from the interRAI HC as the questions are recorded from the perspective of the patient or caregiver but are consistent with the content of items from the interRAI HC. It is embedded with a number of decision support risk algorithms that assist with care planning and service ordering that are also consistent with the interRAI HC and other interRAI assessment tools to support continuity of care across time and sectors [14,15]. Care coordinators use the algorithm scales and outputs, along with clinical judgement, to design person-centred care plans that include various health services and community supports. During the study period, the results of the CU were also used to ascertain if the patient required a more comprehensive clinician-administered assessment.

The impact of the use of the interRAI CU on care planning and care coordination decisions has not been examined in the context of this shift from face-to-face to video call or telephone assessments. Therefore, this study examines the perspectives of care coordinators within the WW and HNHB Home and Community Care Support Services who administered the CU assessment, over the telephone or with video, during the COVID-19 pandemic. We also explored the use of generated interRAI algorithms from the CU in guiding care planning, and whether there were any differences from how these same algorithms were previously used as generated with the interRAI HC. We elected to focus on the Detection of Indicators and Vulnerabilities for Emergency Room Trips (DIVERT) Scale, given reduced primary care visits and reported hesitance to use ED services [16], and the Changes in Health, End-Stage Disease and Symptoms (CHESS) score, which indicates health instability [17]. This work will inform the education and preparation needs of health professionals adopting the CU assessment and the utility and feasibility of conducting non-face-to-face comprehensive assessments.

## Methods

### Study Design

We conducted a mixed-methods study, which involved the collection and analysis of both quantitative and qualitative data. This approach was appropriate to thoroughly answer the research questions and examine multiple facets of the phenomenon using complementary methods [18].

### Data Source

This study’s two data sources were a cross-sectional survey and focus groups.

#### Setting

This study was conducted in two home care regions in Ontario, Canada. Home and Community Care Support Services cover a population of approximately 2 million residents in HNHB and WW regions [19]. The home care population who had the CU administered were long-stay patients. For these individuals the CU may have been conducted as an initial assessment or as a reassessment during the study time period. For the purpose of understanding service intensity needs and acuity of long-stay patients, the following subpopulation categories are used within the Ontario home care sector: community independence, community chronic and community complex. Subpopulations are categorized according to the Client Care Model; community independence is defined as having moderate to low case management intensity with RAI assessment score of 1–10 and stable support network, community chronic is defined as requiring moderate case management intensity with RAI assessment score of 11–16 and one or more chronic conditions with complicating factors, and community complex is defined as requiring high intensity case management and system navigation with a RAI assessment score of 17+ and complex medical, physical, cognitive and social conditions with complicating factors [20].

### Sample

#### Inclusion Criteria

The sample consisted of care coordinators of various health professional backgrounds (e.g., nursing, social work) who worked in the province of Ontario, Canada, and implemented the interRAI CU over the telephone or with video at least once between March 2020 to September 2021. Our data collection instruments limited our sample to those who could read and speak English.

#### Sampling Strategy

Survey participants were recruited using convenience sampling, which involved recruiting participants who met the study eligibility criteria and were accessible to the research team. Eligible individuals working in the WW and HNHB Home and Community Care Support Services regions were contacted via email and provided a link to the survey. At the time of survey distribution, the CU had been in use for at least six months, care coordinators were able to complete the survey once, thereby representing each care coordinator’s cumulative experience using the CU. The final question in the survey invited participants to the focus groups by asking interested individuals to provide their contact information. Study participants for the survey and focus group were not reimbursed.

#### Sample Size

We did not pre-specify a target sample size or compute any power calculations due to the descriptive nature of this survey. We were eager to hear from as many participants as possible while recognizing the ongoing challenges of the COVID-19 pandemic (e.g., the strain on health professionals and survey fatigue). For the focus groups, we aimed to conduct three to five focus groups with four to six participants to ensure representation of different regional contexts and health professional designations.

### Data Collection

#### Survey

We developed a survey consisting of 27 questions, including both structured questions (e.g., multiple-choice and ranking/scale questions) and free-text responses. The survey included questions about implementing the CU with patients, followed by questions specific to completing the CU over the phone and finally applying results of the CU for care planning and specific use of interRAI generated risk algorithm scores. See supplement for survey questions. Qualtrics was used to design and distribute the survey. The survey aimed to gather opinions and perspectives about the implementation, feasibility, and utility of administering the interRAI CU over the telephone or with video in home care settings. The survey was developed in conjunction with the site and reviewed by an interRAI scientist before finalization.

#### Focus Groups

The focus group guide consisted of 12 questions regarding care coordination during the pandemic (*Describe the experience of Care Coordination during the Pandemic*), population contextual factors (*Were there any groups of patients for whom a phone assessment using the CU was done but needed further assessment?*), language and phrasing of CU questions (*Was the language and phrasing of the questions appropriate for most patients/clients? Please elaborate*.), modes of assessment delivery (*face-to-face, virtual, telephone*), the utility for care planning, and use of scales (*How did you use the embedded decision support tools, how did this compare to previous practice?*). The focus group questions aligned with the survey questions and provided an opportunity for further exploration and explanation. We conducted the focus groups online via Microsoft Teams lasting approximately 60 minutes. The focus groups were audio-recorded and transcribed verbatim by the research team.

### Analytic Approach

#### Quantitative Data Analysis

Survey data were pooled and analyzed using descriptive statistics to produce frequency measures in Statistical Analysis Software (SAS version 9.4). The analysis included counts and proportions to summarize responses. Data were presented using tables, where applicable. We stratified some measures based on the care coordinators’ years of experience and designated health profession.

#### Qualitative Data Analysis

The research team used thematic analysis as the primary approach to analyze focus group transcripts and free-text survey responses. The thematic analysis involved identifying patterns in participants’ thoughts, feelings, and practices and drawing interpretations from those patterns – using the steps outlined by Braun and Clarke [21]. Codebooks were created for each data source to assign initial codes and establish categories. We conducted the analysis in pairs to ensure consistency in our interpretation of the data. The researchers frequently referenced the project’s research questions to ensure their analysis was targeted.

#### Mixing of the Data

Data collected in some survey questions was matched with related exploratory focus group questions for triangulation of results [22,23].

### Ethics

This study received ethics approval from Brock University in August 2021 (21–038). For the survey, voluntary participation implied informed consent when entering the Qualtrics survey. Prior to beginning the survey, verbiage was provided that by proceeding with the survey, participants were consenting to collection of their anonymous responses. Written consent was obtained from focus group participants prior to participating. The informed consent form for focus groups included a statement explaining the purposes of the research, describing how information was collected and used, indicating confidentiality measures, and clarifying the voluntary nature of participation. All data were de-identified and aggregated to ensure anonymity. Data analyses were conducted on secure Statistical Analysis Software and NVivo Software, and only the PI and Research Assistant had access to the raw data.

## Results

Forty-eight care coordinators working in the HNHB (n = 22) and WW (n = 26) Home and Community Care Support Services regions completed our survey (Table 1). Most respondents were Registered Nurses (n = 31), while some were Social Workers (n = 8) or Allied Health Professionals (Therapists) (n = 9). All care coordinators had administered the CU at least once over the telephone or with video during the COVID-19 pandemic, and most had also administered the interRAI HC (n = 44; 91.7%).

**Table 1:** Survey respondents’ demographics.


	N (%)

**Gender**	

Female	48 (100.0%)

**Age**	

Less than 40 years old	15 (31.3%)

40 to 49 years old	18 (37.5%)

50 years and older	15 (31.3%)

**Experience as a Care Coordinator**	

Less than 5 years	17 (35.42%)

5 years to less than 10 years	16 (33.3%)

10 years or greater	15 (31.25%)

**Designated Health Profession**	

Registered Nurse (RN)	31 (64.6%)

Social Worker (SW)	8 (16.7%)

Therapies	9 (18.75%)


HNHB = Hamilton Niagara Haldimand Brant; WW = Waterloo Wellington. Therapies = Occupational Therapist, Physiotherapist, and Speech Language Pathologist.

A total of seven survey respondents consented to participate in a focus group, which were offered over three dates to accommodate participant availability. Three one-hour focus groups were conducted each with 2–3 participants. Focus group participants’ experiences as a care coordinator ranged from two to seven years, with professional designations of registered nurse, occupational therapist, or social worker represented. Select quotes from the focus groups are identified by focus group (FG) followed by a participant number.

We report the findings from our survey and focus groups by the main research objectives:

### Objective 1 – Perspectives on implementing the CU over the telephone or virtually

The CU tended to take at least thirty minutes to administer and was most frequently completed for community independent or chronic patient populations (Table 2). Patients were the primary respondents of the CU, although caregivers aided in providing some responses. There was no clear consensus about whether care coordinators identified and prioritized patients needing a more comprehensive assessment based on the CU results. Most care coordinators reported that the CU completed over the telephone “sometimes,” “most times,” or “always” provided a sufficient patient profile for care planning.

**Table 2:** Care Coordinators’ use of the CU.


	N (%)

**Time to administer the CU during a telephone assessment** ^a^	

<20 minutes	3 (6.3%)

20–29 minutes	8 (16.7%)

30–39 minutes	14 (29.2%)

40–49 minutes	13 (27.1%)

>50 minutes	10 (20.8%)

**Patient populations that the CU was administered for**	

Community independence	47

Chronic – initial	13

Chronic – reassessment	29

Complex – initial	2

Complex – reassessment	6

Patients with cognitive impairment (or their caregivers)	6

Long-term care – reassessment	3

**Primary respondent** ^b^	

Caregiver	4 (8.3%)

Caregiver (primarily) and the patient provided some responses	11 (22.9%)

The caregiver and patient equally	6 (12.5%)

Patient (primarily) and the caregiver provided some responses	17 (35.4%)

Patient	10 (20.8%)

**Based on the CU results, how often Care Coordinators identified and prioritized patients needing a more comprehensive assessment**	

Always	6 (12.5%)

Most of the time	9 (18.8%)

Sometimes	17 (35.4%)

Rarely	9 (18.8%)

Never	4 (8.3%)

No response (blank)	3 (6.3%)

**How often the CU completed over the phone provides a sufficient picture of the patient to create a care plan**	

Always	6 (12.5%)

Most of the time	17 (35.4%)

Sometimes	15 (31.3%)

Rarely	6 (12.5%)

Never	1 (2.1%)

No response (blank)	3 (6.3%)


HNHB = Hamilton Niagara Haldimand Brant; WW = Waterloo Wellington. ^a^ On average. ^b^ Most often. ^c^ Such as when the example provided in the assessment did not apply to the person.

When asked about limitations encountered completing the CU over the telephone, the most frequently reported were: the inability to see the living environment (n = 35), hearing impairments (n = 34), inability to observe non-verbal responses or cues (n = 33), language barriers (n = 19), difficulty building rapport (n = 17), difficulty understanding the patient (n = 16), and being unable to speak with family members, caregivers, or patients simultaneously (n = 16). These limitations were also evident in the focus group discussions. One participant shared, “the actual seeing of the home piece, sometimes in more complex patients, I felt like we were really missing a lot from perhaps what we would learn from the family” (F3,2). Others “felt [like it was] hard to know if they [met] all of the criteria, because those are the exact things that I felt we needed to feel like those results were valid and reliable” (FG 3,1). Care coordinators expressed these limitations in contrast to typical conditions experienced during a face-to-face assessment.

Care coordinators reported the following advantages of completing the CU over the telephone: no travel time (n = 42), decreased potential spread of COVID-19 (n = 40), efficiency to complete versus the interRAI HC (n = 38), ease of scheduling (n = 34), completing more assessments per day (n = 34), patient preferred telephone assessments (n = 20), and having the outputs available for care planning (n = 18).

#### Feasibility and utility of administering the CU

Care coordinators most frequently reported being “sometimes” or “rarely” uncertain about the person’s response to CU prompts (Table 3). Many assessors provided alternate examples for CU questions, and most care coordinators referred to the patient’s history or other assessments before administering the CU. None of these findings greatly differed by their years of experience as a care coordinator or designated health professional.

**Table 3:** Barriers to administering the CU.


	EXPERIENCE AS A CARE COORDINATOR			DESIGNATED HEALTH PROFESSION		
	
	LESS THAN 5 YEARS	5 YEARS TO 10 YEARS	10 YEARS OR GREATER	RN	SW	THERAPIES

**How often the assessor was uncertain about the person’s response to questions and documented these potential discrepancies**						

All of the time	0 (0.0%)	0 (0.0%)	0 (0.0%)	0 (0.0%)	0 (0.0%)	0 (0.0%)

Most of the time	0 (0.0%)	2 (4.2%)	2 (4.2%)	3 (6.3%)	1 (2.1%)	0 (0.0%)

Sometimes	4 (8.3%)	6 (12.5%)	6 (12.5%)	11 (22.9%)	2 (4.2%)	3 (6.3%)

Rarely	8 (16.7%)	7 (14.6%)	5 (10.4%)	13 (27.1%)	4 (8.3%)	3 (6.3%)

Never	5 (10.4%)	1 (2.1%)	2 (4.17%)	4 (8.3%)	1 (2.1%)	3 (6.3%)

**How often alternate examples for questions were provided**						

All of the time	1 (2.1%)	0 (0.0%)	0 (0.0%)	0 (0.0%)	1 (2.1%)	0 (0.0%)

Most of the time	6 (12.5%)	6 (12.5%)	3 (6.3%)	11 (22.9%)	2 (4.2%)	2 (4.2%)

Sometimes	4 (8.3%)	7 (14.6%)	10 (20.8%)	14 (29.2%)	3 (6.3%)	4 (8.3%)

Rarely	5 (10.4%)	3 (6.3%)	2 (4.2%)	6 (12.5%)	2 (4.2%)	2 (4.2%)

Never	1 (2.1%)	0 (0.0%)	0 (0.0%)	0 (0.0%)	0 (0.0%)	1 (2.1%)

**How often the Care Coordinator accessed the patient’s history or other information before administering the CU**						

All of the time	11 (22.9%)	6 (12.5%)	7 (14.6%)	13 (27.1%)	7 (14.6%)	4 (8.3%)

Most of the time	5 (10.4%)	7 (14.6%)	4 (8.3%)	11 (22.9%)	1 (2.1%)	4 (8.3%)

Sometimes	1 (2.1%)	2 (4.2%)	4 (8.3%)	7 (14.6%)	0 (0.0%)	0 (0.0%)

Rarely	0 (0.0%)	1 (2.1%)	0 (0.0%)	0 (0.0%)	0 (0.0%)	1 (2.1%)

Never	0 (0.0%)	0 (0.0%)	0 (0.0%)	0 (0.0%)	0 (0.0%)	0 (0.0%)


RN = Registered Nurse; SW = Social Worker. Therapies = Occupational Therapist, Physiotherapist, and Speech Language Pathologist.

Qualitative results confer that care coordinators found the CU easy to “pivot” to, given similarities and alignment with the interRAI HC. The CU was considered shorter and saved time, and many care coordinators were comfortable implementing it as an assessment tool over the telephone. The CU was completed primarily over the telephone, while the interRAI HC was typically used for video assessments. Care coordinators indicated that practice recommendations were to use the interRAI HC for video assessments and the CU for telephone assessments. Care coordinators indicated a preference to use the interRAI HC for complex patient populations.

Care coordinators stated a high comfort level communicating with patients and families over the telephone, attributing this to routine practice where communication with patients and family involved follow-up and check-ins completed over the telephone. Care coordinators felt that most patients were “comfortable” answering questions over the telephone, and found their patients appreciative of the interaction.

Qualitative findings derived from the survey free-text option revealed that most commonly reported barriers related to administering the CU, including individual patient characteristics and the format of questions. Care coordinators indicated that the self-report style of questions was “awkward” compared to the more “conversational” flow of the interRAI HC. The survey style of questions and their fixed sequence were the primary reasons cited. An example of this as described by FG1,2: “I just remember very vividly speaking with someone that I had not developed a therapeutic relationship with, and you know, right off the bat, I am asking them if they are sad, or anxious or worried … I think some people are uncomfortable with that.”

### Objective 2 – Use of interRAI scales and outputs generated from the CU

Care coordinators frequently reported that the CU results were “often” or “very often” helpful in gaining an understanding of the patient’s medical issues, functional status, mood, and informal supports (Table 4). Most often, the CU results were “sometimes” helpful in gaining an understanding of the patient’s cognition or social isolation.

**Table 4:** Care Coordinators’ use of CU results for care planning.


HOW OFTEN THE CU RESULTS…	N (%)					

	MEDICAL ISSUES	FUNCTIONAL STATUS/ADLS	MOOD	COGNITION	SOCIAL ISOLATION	INFORMAL SUPPORTS/IADLS

**Help gain an understanding of the patient’s:**						

Very often	3 (6.3%)	9 (18.8%)	4 (8.3%)	5 (10.4%)	5 (10.4%)	5 (10.4%)

Often	15 (31.3%)	25 (52.1%)	20 (41.7%)	16 (33.3%)	16 (33.3%)	29 (60.4%)

Sometimes	21 (43.8%)	9 (19.8%)	14 (29.2%)	18 (37.5%)	17 (35.4%)	9 (18.8%)

Rarely	5 (10.4%)	2 (4.2%)	7 (14.6%)	5 (10.4%)	5 (10.4%)	1 (2.1%)

Never	1 (2.1%)	0 (0.0%)	0 (0.0%)	1 (2.1%)	2 (4.2%)	1 (2.1%)

No response (blank)	3 (6.3%)	3 (6.3%)	3 (6.3%)	3 (6.3%)	3 (6.3%)	3 (6.3%)

**Help in the development of care planning related to:**						

Very often	1 (2.1%)	9 (18.8%)	3 (6.3%)	4 (8.3%)	3 (6.3%)	8 (16.7%)

Often	18 (37.5%)	23 (47.9%)	15 (31.3%)	16 (33.3%)	15 (31.3%)	23 (47.9%)

Sometimes	14 (29.2%)	10 (20.8%)	17 (35.4%)	17 (35.4%)	20 (41.7%)	11 (22.9%)

Rarely	10 (20.8%)	3 (6.3%)	9 (18.8%)	7 (14.6%)	5(10.4%)	2 (4.2%)

Never	2 (4.2%)	0 (0.0%)	1 (2.1%)	1 (2.1%)	2 (4.2%)	1 (2.1%)

No response (blank)	3 (6.3%)	3 (6.3%)	3 (6.3%)	3 (6.3%)	3 (6.3%)	3 (6.3%)


ADLs = Activities of daily living; IADLs = Instrumental activities of daily living.

Care coordinators indicated that certain patient characteristics impede the ability to complete self-report questions, such as cognitive impairments affecting the patient’s insight about their capabilities and needs. As one participant shared,

“The times where it was challenging for me to do, the shift was things around like medications or people who had very little family supports like not being able to see the person and see their blister pack. And if they are kinda taking their medications when you don’t have, you know, maybe family members calling about those kind of high risk people. […] I found the check-ups were really helpful for getting a lot of information on the telephone where there’s a lot of times we’re out on a visit and, you know the person has supports and they’re doing okay, and it’s it is a lot to go out to someone’s home and travel and spend that time just to kind of gather really simple assessment information for sure” (FG 2, 2).

Care coordinators expressed uncertainty regarding the validity of the patient’s answers, which were more pronounced if the patient was new or not previously known to the care coordinator. Care coordinators found the CU useful for patients considered stable, independent, and with minimal care needs. The CU was preferred for reassessments or simple initial assessments, whereas the interRAI HC was viewed as the preferred assessment for complex populations. Care coordinators reported understanding which patient population was most appropriate to use the CU.

Most care coordinators did not use the interRAI DIVERT or CHESS scale scores that were derived from the CU for care planning – regardless of their years of experience or designated health profession (Table 5).

**Table 5:** Care Coordinators’ use of scales for care planning (n = 45).


	EXPERIENCE AS A CARE COORDINATOR			DESIGNATED HEALTH PROFESSION		
	
	LESS THAN 5 YEARS	5 YEARS TO 10 YEARS	10 YEARS OR GREATER	RN	SW	THERAPIES

**How often the DIVERT scale score was used**						

With all patients	2 (4.4%)	2 (4.4%)	0 (0.0%)	0 (0.0%)	1 (2.2%)	3 (6.7%)

With those who were DIVERT 3 or more	1 (2.2%)	0 (0.0%)	1 (2.2%)	1 (2.2%)	0 (0.0%)	1 (2.2%)

With those who were DIVERT 5 or 6	3 (6.7%)	4 (8.9%)	2 (4.4%)	8 (17.8%)	0 (0.0%)	1 (2.2%)

Do not use	11 (24.4%)	8 (17.8%)	11 (24.4%)	19 (42.2%)	7 (15.6%)	4 (8.9%)

**How often the CHESS scale score was used**						

With all patients	3 (6.7%)	4 (8.9%)	2 (4.4%)	4 (8.9%)	2 (4.4%)	3 (6.7%)

With those who scored 3 or more	0 (0.0%)	1 (2.2%)	0 (0.0%)	0 (0.0%)	0 (0.0%)	1 (2.2%)

With those who scored 4 or more	3 (6.7%)	2 (4.4%)	4 (8.9%)	7 (15.6%)	1 (2.2%)	1 (2.2%)

With those who scored End-Stage	1 (2.2%)	1 (2.2%)	0 (0.0%)	2 (4.4%)	0 (0.0%)	0 (0.0%)

Do not use	10 (22.2%)	6 (13.3%)	8 (17.8%)	15 (33.3%)	5 (11.1%)	4 (8.9%)


N = Registered Nurse; SW = Social Worker; OT = Occupational Therapist; PT = Physiotherapist; SLP = Speech Language Pathologist.

In contrast, care coordinators participating in the focus groups indicated that they do review the scales, but intuitively “knew” which patients were at risk, rationalizing their experience completing multiple assessments and understanding the assessment questions. “I will always look at them, but I think it’s mostly the level of care that I really do focus on” (FG2,1). The care planning process is continuous as one completes the assessment, and the algorithm scores add evidence to support care-planning decisions at the end. “Essentially, you are care-planning as you go. When you get to those sections of the RAI Check-Up, you know when you are asking around falls … there are three falls … you’re going to make care planning changes or changes to the services” (FG1, 1).

The CU enabled the care coordinator to “order the appropriate services and refer to community partners” (survey free text) and provided a clearer understanding of how the patient was managing compared to the previous practice of completing functional assessments.

### Care Coordination during the COVID-19 Pandemic

Since the CU assessment was implemented in Ontario during the COVID-19 pandemic, there were several findings related to care coordination and care-planning within this context. The CU provided a means to collect assessment information while ensuring reduced in-person contact to mitigate COVID-19 transmission. At the beginning of the pandemic, “we were at the point where we were just delaying assessments, our hands were completely tied with what we were able to support with” (FG3,2). Compared to the interRAI Contact Assessment, an initial screening assessment used at intake, the CU included more questions and was felt to provide more information to support care planning and care coordination activities. “I think during the pandemic it really highlighted [our role], you know primary care visits were not happening, and sometimes you reaching out with the telephone assessment might have been their only contact with a health care professional” (FG 3,1). The CU was viewed as a timely and necessary assessment tool that ensured continuity of home and community care supports and services during the pandemic.

## Discussion

Our mixed-methods assessment of the interRAI CU tool is one of the first efforts to evaluate its utility in community-based settings, particularly in the context of the COVID-19 pandemic. Care coordinators regarded the CU favourably in providing sufficient information to create a care plan and reported several advantages to completing the CU over the telephone or video call, such as reduced travel time, risk of disease transmission, and time spent administering the assessment. These advantages affirm the efficiency of conducting video/telephone assessments, especially during infectious disease outbreaks (e.g., COVID-19, influenza). However, limitations associated with telephone assessments were noted, including being unable to assess the living environment, communication barriers with respondents, and the inability to notice non-verbal cues.

Our findings indicate that telephone and video assessments were not feasible for some patient populations, such as those with cognitive impairment, since care coordinators expressed concerns that these patients lacked insight about their own needs and risks. Similar findings have been shown comparing ability to complete video versus phone assessments in ambulatory care for older patients [24]. Utilization barriers among older adults, including visual and auditory sensory deficits, technical literacy, mental acuity, and lack of technical support have been previously documented [25]. Additional considerations, include the social and economic determinants of health that would influence one’s ability to engage with the health provider and access required technology, such as a telephone [26]. Characteristics of the population must be considered when introducing a comprehensive video or phone assessment to ensure marginalization is avoided and reliable assessment data is obtained [27]. For some of these identified barriers, the CU offers the flexibility of the caregiver responding on behalf of the patient.

Although care coordinators in our study tended to leverage previous medical or assessment information to inform the assessment, they did so more when the patients were unknown to them (e.g., initial assessments). During the time of this study elderly individuals struggled with delayed or missed care for chronic conditions, contributing to poorer health outcomes and the availability of current health information [28]. Specific concerns voiced by the care coordinators included the self-reported nature of the assessment, as care coordinators faced difficulty ascertaining or trusting the results to determine an appropriate care plan. Accordingly, care coordinators believe the interRAI HC was the ideal assessment tool for clients with complex needs, whereas the CU was a suitable reassessment tool for clients. The suite of interRAI assessments are designed for use with specific populations, the home care setting with it’s variation in patient complexity is ideal for a stepped approach to assessment using both the CU and RAI HC [29]. Despite the large influx of virtual care, many elderly individuals still reported a lack of access to home care services [28]. As the aging population continues to grow, it is important to evaluate the availability and efficiency of home care assessments.

Care coordinators indicated that they felt prepared to administer the CU assessment and that the orientation provided was adequate. It is important to note that the interRAI suite of assessments contain questions that align across the assessments, so that health information is integrated and can be compared to previous assessments. Familiarity with the interRAI Home Care assessment may have made the switch to the CU easy, given previous use. Adoption of the CU, as a new assessment instrument did not elicit staff resistance, as previously seen with the introduction of similar practice change [30,31]. Related, the assessment tool used at the beginning of the pandemic (i.e., functional assessment template) was not structured or configured to interface with other interRAI assessments or generate risk algorithms, decreasing its utility, as perceived by the care coordinators in our study. Clinicians have previously reported benefits using the interRAI risk algorithms and clinical assessment protocols to guide practice and care decisions [32,33,34]. Assessors were comfortable communicating with informal caregivers while completing the CU, which may stem from ongoing/routine contact to assess clients’ changing needs and updates to their care plan. Overall, care coordinators believe the CU offered an assessment that “fit” with other interRAI assessments used for care coordination.

Limitations of our study include that adoption of the CU occurred under extenuating circumstances, where routine in-home assessments were not being completed due to public health mandates [8,35]. We aimed to better understand how care coordinators use the interRAI scales and outputs, however there is a paucity in the literature for comparison. Our study was limited to Ontario regions where the CU was implemented; results from this convenience sample of care coordinators may not be generalizable to care coordination practice in other provinces or countries. Care coordinators who participated in this study were also familiar with interRAI instruments (ie. interRAI HC), additional training and education for assessors without experience using interRAI assessments would be required. Of note, the CU has been successfully implemented in community settings with non-health professionals administering the assessment [15]. Care coordinators indicated that the CU was well received and provided a means to perform care coordination activities and resume day-to-day practice.

## Conclusion

Our findings support the use of the interRAI CU, a comprehensive self-report assessment, as a promising addition to the suite of tools for home care coordination. Care coordinators regarded the CU favourably in providing sufficient information to create a care plan and reported several advantages to completing the assessment with video or over the phone, such as reduced travel time, risk of disease transmission, and time spent administering the CU. These advantages affirm the efficiency of conducting virtual assessments, particularly during periods when face-to-face contact is not advised (e.g., COVID-19 or influenza outbreaks).

Our work highlights strengths and limitations in the use of the CU, which may be informative as additional compendium and training documents are developed. Limitations of the CU, when completed virtually, included not being able to assess the patient’s living environment, communication barriers with respondents, and the inability to pick-up on non-verbal cues. These barriers were most evident with patients who were unknown to the care coordinator (i.e., at initial assessment) and when cognitive status was unclear. Additionally, care coordinators believe the interRAI HC was the ideal initial assessment tool for patients with complex needs, whereas the CU was a suitable reassessment tool for these patients. Assessors were often comfortable communicating with informal caregivers while completing the CU, which may stem from routine contact with patients/families to assess patient’s changing needs and update care plans. Future studies should include delineating patient populations and conditions most appropriate for adoption and education to reinforce the use of interRAI scales and outputs to ensure consistent care planning. Our study suggests that integration of a self-report style comprehensive assessment is feasible and efforts to determine where best the CU would fit in the patient’s care trajectory should be explored. Future work should consider the opportunities and limitations identified in this study when thinking about the CU’s utility and adoption after the pandemic.

## Additional File

The additional file for this article can be found as follows:

10.5334/ijic.7007.s1 Supplement. Check-Up Survey Questions.
