# Supplementary material for: Implementing the interRAI Check-Up Comprehensive Assessment: Facilitating Care Planning and Care Coordination during the Pandemic
Source: Int J Integr Care. 2023 Apr 10;23(2):5. doi: 10.5334/ijic.7007 (PMC10120599; doi:10.5334/ijic.7007)
Supplement: Supplement. — Check-Up Survey Questions. [file ijic-23-2-7007-s1.pdf]

# Supplement - Check-Up Survey Questions

## Demographics:

1. Consent
2. Eligibility Care Coordinator question
3. Role as a CC
4. Experience in years as a CC
5. Health profession designation
6. Gender
7. Age
8. Region

## Conducting the CU/Telephone Assessment:

Have you administered the **interRAI Check-Up** at least once during a **telephone assessment** in the past six months?

a. Yes
b. No → **Do not proceed with survey [Exit Survey]**

9. On average, how long did it take you to administer the Check-Up during a telephone assessment?
   c. <20 minutes
   d. 20-29 minutes
   e. 30-39 minutes
   f. 40-49 minutes
   g. >50 minutes

10. Do you feel you understand which population to use the Check Up for? [Comment Box]

11. What patient populations did you use the Check-Up for? Select all that apply.
   h. Community Independence
   i. Chronic Initial
   j. Chronic Reassessment
   k. Complex Initial
   l. Complex Reassessment
   m. Patients with cognitive impairment (or their caregivers)
   n. Long-term Care Reassessment

12. When conducting the Check-Up Assessment, who was the primary respondent most often?
   o. The caregiver often provided all the requested information
   p. The caregiver provided most of the requested information, but the patient provided some responses

q. The caregiver and patient equally provided the requested information
r. The patient most of the requested information, and the caregiver provided some responses
s. The patient often provided all the requested information

13. On average, how often did you provide alternate examples for questions, such as when the example provided did not apply to the person?
    t. All of the time
    u. Most of the time
    v. Sometimes
    w. Rarely
    x. Never

14. On average, how often did you have access to the patient history or other patient assessments/information prior to the Check Up assessment?
    y. All of the time
    z. Most of the time
    aa. Sometimes
    bb. Rarely
    cc. Never

15. On average, how often were you uncertain about the person's response to questions and document these potential discrepancies?
    dd. All of the time
    ee. Most of the time
    ff. Sometimes
    gg. Rarely
    hh. Never

16. Identify any limitations encountered completing the assessment over the phone. Please select all that apply.
    a. Difficulty understanding patient
    b. Hearing impairment
    c. Language barrier
    d. Unable to speak to family/caregiver and patient at same time
    e. Unable to see living environment
    f. Inability to see/pick up on non-verbal responses
    g. Difficulty building rapport
    h. Other
    [Comment Box]

17. Identify the advantages of completing the assessment over the phone. Please select all that apply.
    a. Saved travel time

b. Easier to schedule/client availability
c. Took less time to complete than the interRAI-HC
d. Can assess more patients in one day
e. Decreased potential spread of COVID 19
f. Patient preference for telephone assessment
g. Outputs available to use for care planning (compared to template or Functional Assessment)
h. Other
[Comment Box]

18. Have you done the interRAI HC virtually?
a. Yes
b. No

19. If yes, how does the interRAI CU compare? [Comment Box]

## Interpreting the results and applying for service planning:

20. For the majority of patients, how often did the Check-Up results help you gain an **understanding** of _____________?

| | Never | Rarely | Sometimes | Often | Very often |
|---|---|---|---|---|---|
| Medical Issues | | | | | |
| Functional status/ ADLs | | | | | |
| Mood | | | | | |
| Cognition | | | | | |
| Social Isolation | | | | | |
| Informal Supports IADLs | | | | | |

21. For the majority of patients, how often did the Check-Up results help **in the development of care planning** related to _____?

| | Never | Rarely | Sometimes | Often | Very often |
|---|---|---|---|---|---|
| Medical Issues | | | | | |
| Functional Status ADLs | | | | | |
| Mood | | | | | |
| Cognition | | | | | |
| Social Isolation | | | | | |
| Informal Support IADLs | | | | | |

22. Based on the Check-Up results, how often did you **identify and prioritize patients needing a more comprehensive assessment (RAI- HC)**?
ii. Always

jj. Most of the time
kk. Sometimes
ll. Rarely
mm. Never

23. Does the Check-Up completed over the phone **provide a sufficient picture of the client** to create a care plan?

nn. Always
oo. Most of the time
pp. Sometimes
qq. Rarely
rr. Never

[Comment Box]

**We are interested in learning more about how you may have used the DIVERT Scale and CHESS Score.**

24. How often did you use the DIVERT Scale?
    a. With all patients
    b. With those who were DIVERT 3 or more
    c. With those who were DIVERT 5-6
    d. Do not use
25. If used, how did you use the DIVERT Scale? [Comment Box]

26. How often did you use the CHESS Score?
    a. With all patients
    b. With those who scored 3 or more
    c. With those who scored 4 or more
    d. With those who scored End Stage
    e. Do not use
27. If used, how did you use the CHESS scale? [Comment Box]
